# Mechanically Adaptive Mixed Ionic-Electronic Conductors Based on a Polar Polythiophene Reinforced with Cellulose Nanofibrils

**DOI:** 10.1021/acsami.3c03962

**Published:** 2023-06-01

**Authors:** Mariza Mone, Youngseok Kim, Sozan Darabi, Sepideh Zokaei, Lovisa Karlsson, Mariavittoria Craighero, Simone Fabiano, Renee Kroon, Christian Müller

**Affiliations:** † Department of Chemistry and Chemical Engineering, Chalmers University of Technology, 412 96 Göteborg, Sweden; ‡ Wallenberg Wood Science Center, Chalmers University of Technology, 412 96 Göteborg, Sweden; § Laboratory of Organic Electronics, Department of Science and Technology, Linköping University, 602 21 Norrköping, Sweden; ∥ Wallenberg Wood Science Center, Linköping University, 602 21 Norrköping, Sweden

**Keywords:** cellulose nanofibrils (CNF), organic mixed ionic-electronic conductors, conjugated polymer, organic electrochemical transistor (OECT), chemical doping

## Abstract

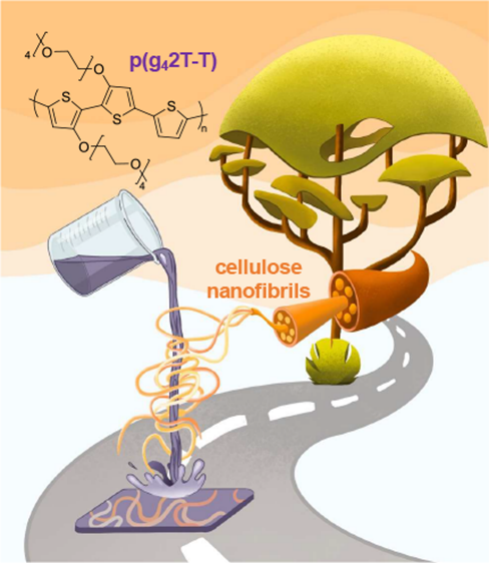

Conjugated polymers with oligoether side chains are promising mixed ionic-electronic conductors, but they tend to feature a low glass transition temperature and hence a low elastic modulus, which prevents their use if mechanical robust materials are required. Carboxymethylated cellulose nanofibrils (CNF) are found to be a suitable reinforcing agent for a soft polythiophene with tetraethylene glycol side chains. Dry nanocomposites feature a Young’s modulus of more than 400 MPa, which reversibly decreases to 10 MPa or less upon passive swelling through water uptake. The presence of CNF results in a slight decrease in electronic mobility but enhances the ionic mobility and volumetric capacitance, with the latter increasing from 164 to 197 F cm^–3^ upon the addition of 20 vol % CNF. Overall, organic electrochemical transistors (OECTs) feature a higher switching speed and a transconductance that is independent of the CNF content up to at least 20 vol % CNF. Hence, CNF-reinforced conjugated polymers with oligoether side chains facilitate the design of mechanically adaptive mixed ionic-electronic conductors for wearable electronics and bioelectronics.

## Introduction

Conjugated polymers are widely studied for optoelectronic applications ranging from organic photovoltaics and thermoelectrics to bioelectronics.^1−3^ The type and number of side chains strongly impact the solubility, solid-state order as well as the ability to transport ions and electronic charge.^4,5^ Alkyl and oligoether side chains are widely used, and both types facilitate solubility of conjugated polymers in organic solvents, with the latter ones enabling processing from solvents of higher polarity.^6^ Conjugated polymers with oligoether side chains can undergo significant passive swelling, i.e., the uptake of water when submerged in an aqueous electrolyte, as well as active swelling, i.e., the ingression of additional electrolyte upon the application of an electrochemical potential.^7,8^ Conjugated polymer films display mixed ionic and electronic conduction, i.e., the ability to transport both ions and electronic charge carriers, which is of considerable interest for bioelectronics and exploited in devices such as organic electrochemical transistors (OECTs) and ion pumps.^9−11^

**Figure 1:**
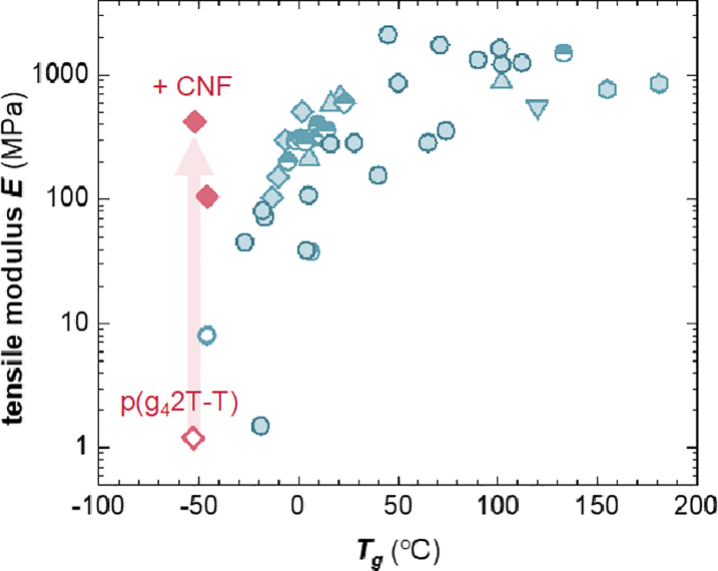
Tensile elastic modulus at room temperature vs the *T*_g_ of p(g_4_2T-T) from ref (12) (blue open circle), of conjugated polymers with alkyl side chains from refs (22−27) (blue filled symbols) and refs (28, 29) [blue half-filled symbols; tensile elastic modulus estimated using *E* = 2*G*(1 + *υ*), where *G* is the shear elastic modulus and assuming a Poisson ratio of *υ* = 0.5] as well as the value measured in this study for p(g42T-T) (pink open diamond), which increases by at least two orders of magnitude (red arrow) upon the addition of 11 or 20 vol % CNF (pink filled diamonds).

**Figure 2:**
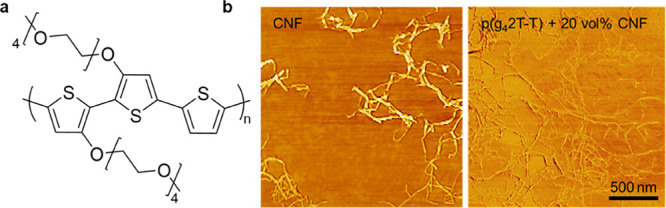
(a) Molecular structure of p(g_4_2T-T). (b) AFM phase images of neat CNF (left) and a 20 nm thin film of a nanocomposite of p(g_4_2T-T) and 20 vol % CNF (right).

Oligoether side chains also endow conjugated polymers with a sub-zero glass transition temperature *T*_g_,^12^ in contrast to polymers with alkyl side chains, which can feature a *T*_g_ considerably above room temperature (Figure 1).^13^ It can be anticipated that a sub-zero *T*_g_ will result in fast backbone as well as side-chain relaxation kinetics at ambient conditions, which benefits ion mobility. On the other hand, a low *T*_g_ also tends to result in a soft material at room temperature, especially if the crystallinity of the polymer is low. Accordingly, conjugated polymers with oligoether side chains display a much lower tensile elastic modulus at room temperature, e.g., *E* < 10 MPa in the case of p(g_4_2T-T) with tetraethylene glycol side chains,^12^ which is the subject of this study (see Figure 2a for the chemical structure), and *E* < 50 MPa in the case of a high-molecular-weight grade of a similar polythiophene with shorter triethylene glycol side chains.^14^ In contrast, polymers that bear alkyl side chains tend to feature values of *E* ≫ 100 MPa, especially when paired with an above room temperature *T*_g_ (Figure 1). Water uptake by polymers with oligoether side chains can be expected to lead to a further reduction in elastic modulus. Such soft materials must be used with an adequate support such as a (flexible) substrate to facilitate their use where mechanical robustness is required, e.g., in the case of implantable electrodes,^15^ conducting fibers for e-textile devices^16,17^ such as fiber-based electrochemical transistors,^18,19^ or the legs of a thermoelectric generator.^1^ For some applications, it would be desirable if the material or device was initially rigid to facilitate, e.g., insertion as a free-standing artifact into a biological tissue but then became soft once installed or implanted.^20,21^

To modulate the mechanical properties of conjugated polymers, strategies such as the introduction of flexible spacers,^30^ copolymerization^31,32^ or blending with an insulating polymer,^33^ compounding with nanocellulose,^34^ and molecular doping^12^ have been explored. Nanocomposites are intriguing because they may allow increasing the stiffness of the material without unduly compromising ion mobility and chain relaxation dynamics within the conjugated polymer matrix. Nanocellulose continues to receive widespread attention as a reinforcing agent for polymers due to its high strength, stiffness, and aspect ratio.^35,36^ Nanocellulose has been used as an additive for a number of different conjugated polymer-based materials such as polypyrrole (PPy),^37,38^ polyaniline (PANI),^34^ and poly(*p*-phenylene ethynylene) (PPE)^34^ as well as poly(3,4-ethylenedioxythiophene):poly(styrene sulfonate) (PEDOT:PSS).^39−41^ For example, the addition of cellulose nanofibrils (CNFs) with a degree of carboxymethyl substitution of up to 0.25 to PEDOT:PSS resulted in composite materials with an electrical conductivity of about 350 S cm^–1^.^40^ Many conjugated polymers, in particular those with alkyl side chains, are soluble in apolar organic solvents, complicating compounding with nanocellulose, which is typically processed from polar solvents such as water or dimethylformamide (DMF).

Here, we study the mechanical and electrochemical properties of nanocomposites composed of the polar polythiophene p(g_4_2T-T) and CNF. The addition of CNF to p(g_4_2T-T) allows us to reinforce the polymer without affecting the *T*_g_ (Figure 1) or the transconductance of nanocomposite-based OECTs. Swelling of p(g_4_2T-T):CNF nanocomposites through water uptake results in a reversible decrease in elastic modulus from *E* > 100 MPa to about 10 MPa or less, which opens up the use of cellulose nanocomposites for the preparation of mechanically adaptive materials for wearable electronics and bioelectronics.

## Results and Discussion

We chose to work with carboxymethylated CNF with a degree of substitution of 0.1, i.e., 10% of the hydroxy groups of cellulose have been replaced with a carboxymethyl group, which has been found to allow the preparation of highly conducting nanocomposites in the case of PEDOT:PSS and CNF.^40^ A predetermined amount of a DMF solution of p(g_4_2T-T) was added to a DMF dispersion of carboxymethylated CNF (see the Experimental Section for details). The mixture was stirred for 1 h and then used to prepare nanocomposite films by either drop casting (thick films) or wire bar coating (thin films). The resulting nanocomposites had a CNF content that varied from 0.6 to 33 vol % assuming densities of 1 and 1.6 g cm^–3^ for p(g_4_2T-T) and CNF, respectively.

Atomic force microscopy (AFM) was used to assess the distribution of CNF in the polymer matrix. Quantitative analysis of the height of neat fibrils revealed a diameter of 4.6 ± 1.1 nm. We were only able to distinguish the nanocellulose in very thin films (thickness *d* ≈ 20 nm) containing at least 20 vol % CNF (Figure 2b). The recorded AFM images indicate that a significant fraction of CNF is present as individual fibrils. The observed fibrils on the film surface had a height of 2.7 ± 0.9 nm, which suggests that they are in part embedded in the polymer matrix.

Dynamic mechanical analysis (DMA) was used to determine the impact of the nanocellulose on the *T*_g_ of the polymer. For neat p(g_4_2T-T), we obtained a *T*_g_ = −53 °C from the peak in loss modulus (Figure S1). For the nanocomposites containing 11 and 20 vol % CNF, two peaks were observed, the first one located at −46 and −52 °C and a second peak at 5 or −3 °C, respectively (Figure S1). The presence of two *T*_g_ values can be attributed to the existence of two types of domains in the polymeric matrix, i.e., a bulk material with a similar *T*_g_ as neat p(g_4_2T-T) and a polymer layer close to the CNF surface where chain motion is restricted, yielding a higher *T*_g_. Note that nanocellulose does not display a glass transition.^42^

Wide-angle X-ray scattering (WAXS) was performed to analyze the crystalline order of neat p(g_4_2T-T) and the nanocomposites containing 3, 11, and 20 vol % CNF (Figure S2). The WAXS diffractogram of neat p(g_4_2T-T) featured two peaks at *q*_100_ ≈ 0.35 Å^–1^ and *q*_200_ ≈ 0.72 Å^–1^, characteristic of lamellar stacking.^43^ At higher wave vectors, only a broad amorphous halo with a peak at *q* ≈ 1.6 Å^–1^ was present but no π-stacking peak could be observed. The addition of CNF results in a considerable decrease of the intensity of the lamellar stacking peaks, which does not scale with the remaining polymer content. Hence, it appears that the presence of CNF disrupts lamellar stacking of the polymer to some extent. Concomitantly, a distinct peak emerges at *q*_200_ ≈ 1.62 Å^–1^ in the case of nanocomposites containing 11 and 20 vol % CNF (Figure S2), which we assign to the 200 diffraction of CNF,^44^ instead of π-stacking of the polymer (which would appear at a similar position) since CNF appears to disrupt its ability to order.

**Figure 3:**
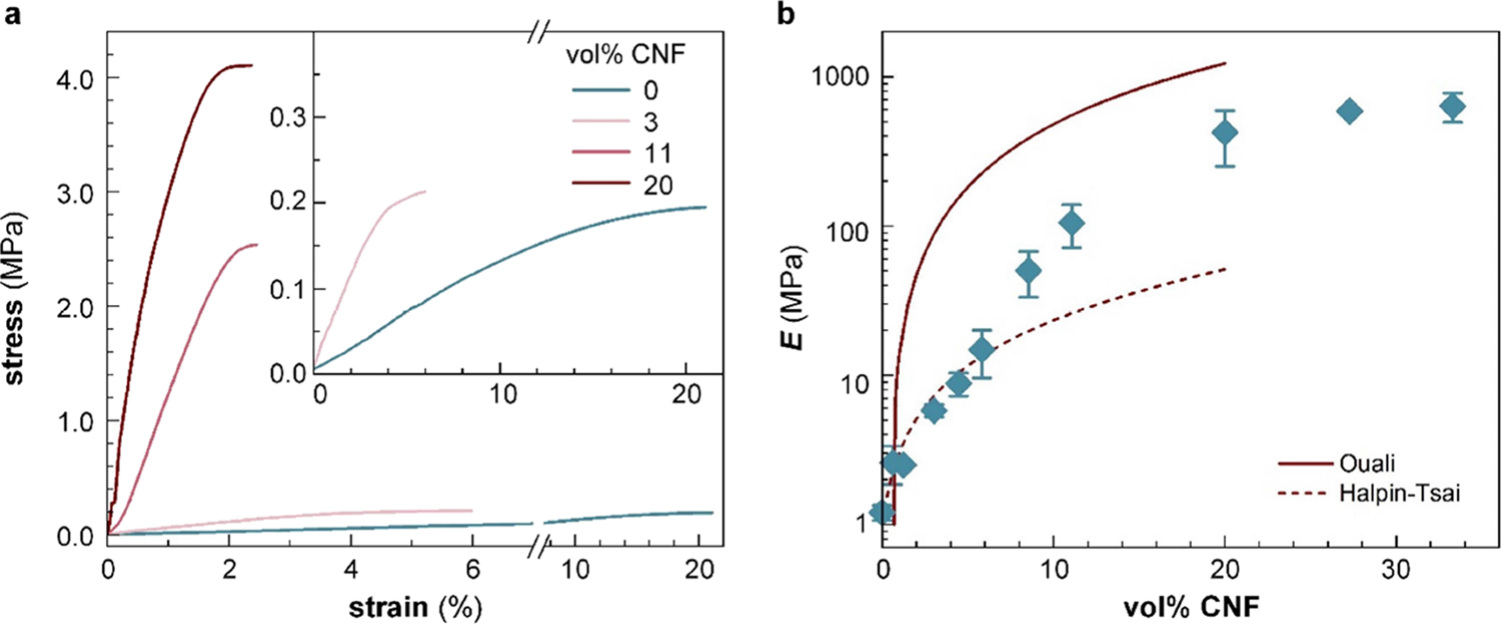
Tensile deformation of dry nanocomposites. (a) Engineering stress vs strain recorded at room temperature by tensile deformation of neat p(g_4_2T-T) (blue) and p(g_4_2T-T):CNF nanocomposites (red). The inset shows the stress–strain curves for neat and 3 vol % CNF nanocomposites. (b) Young’s moduli of p(g_4_2T-T):CNF nanocomposites as a function of the volume percentage (vol %) of CNF. The dashed and solid lines are predictions made using the Halpin-Tsai and Ouali models, respectively. The data points and error bars represent the mean value and min–max error for measurements of three to four individual samples.

We used tensile deformation to compare the mechanical properties of the p(g_4_2T-T):CNF nanocomposites. The Young’s modulus strongly increased upon the addition of CNF from *E*_m_ = 1 MPa for the neat polymer to *E* = 756 MPa for p(g_4_2T-T):CNF containing 27 vol % CNF (Figures 3a, S3 and Table S1). The value of neat p(g_4_2T-T) is lower than a previous measurement (see Figure 1),^12^ which we explain with a lower degree of order of the here used polymer batch. The elongation at break decreased from ε_b_ > 20% for a CNF content of up to 3 vol % to ε_b_ ≲ 10% for larger amounts of CNF. The experimentally obtained Young’s modulus values were compared with those predicted by the Ouali and Halpin-Tsai models, which can be used to describe the reinforcing effect obtained for a percolating network of the reinforcing agent and a material dominated by filler–matrix interactions, respectively.^45−47^

According to the Ouali model, the elastic modulus is given by:^46,47^
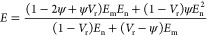
1where *V*_r_ is the volume fraction of the reinforcing agent, *E*_m_ = 1 MPa is the elastic modulus of the matrix, *E*_n_ is the elastic modulus of the reinforcing network, here given a value reported for a microfibrillated cellulose nanopaper, *E*_n_ = 13.2 GPa,^48^ and ψ is the volume fraction of the reinforcing agent that participates in the percolating network:
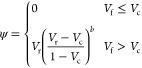
2where *V*_c_ is the percolation threshold (here given a value of 0.7 vol %) and *b* is the critical percolation exponent with a value of 0.4 in the case of a three-dimensional (3D) network.^46^ The modulus predicted by the Ouali model increases rapidly above *V*_c_, significantly exceeding experimental values (Figure 3b), and thus, we argue that a reinforcing filler network is likely not a good description of the here studied p(g_4_2T-T):CNF nanocomposites.

The Halpin-Tsai model allows us to estimate Young’s modulus according to:^45^
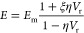
3where ξ/2 is the aspect ratio of the reinforcing agent (here given a value of ξ/2 = 100) and η is given by
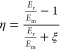
4where *E*_r_ is Young’s modulus of the reinforcing agent, here given the value reported for cellulose I single crystals, i.e., *E*_r_ = 130 GPa.^49^ The increase in modulus predicted by the Halpin-Tsai model matches the experimental values up to 6 vol % CNF (Figure 3b), and thus, we propose that a material dominated by filler:matrix interactions is a reasonable description of p(g_4_2T-T):CNF nanocomposites with a low CNF content. For higher CNF concentrations, the measured Young’s modulus exceeds the values predicted by the Halpin-Tsai model and instead approaches the values predicted by the Ouali model, which we assign to partial aggregation of CNF leading to an increased influence of filler:filler interactions.

**Figure 4:**
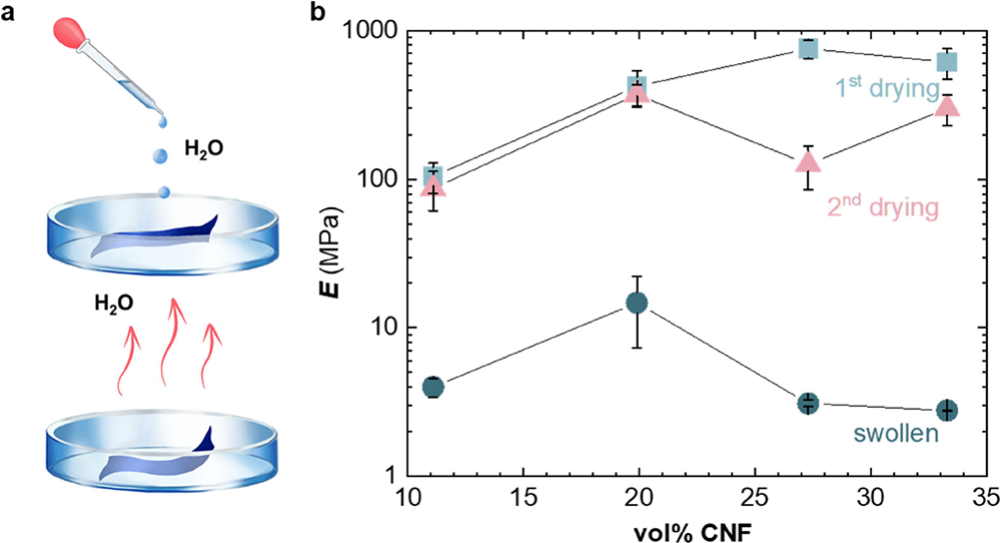
Water uptake. (a) Schematic of the swelling experiments. (b) Young’s moduli of p(g_4_2T-T):CNF nanocomposites as a function of vol % CNF for initial vacuum-dried samples (blue squares), samples swollen in deionized water (blue circles), and the same samples dried again in vacuum (red triangles). Each data point and error bar represent the mean value and min–max error for measurements of two to four individual samples.

In a further set of experiments, we explored the change in mechanical properties of the nanocomposites upon swelling through water uptake. Vacuum oven dried samples of p(g_4_2T-T):CNF nanocomposites with 11 to 33 vol % CNF were immersed in deionized water for approximately 48 h (Figure 4a), immediately followed by tensile deformation since the samples rapidly dried once removed from the water bath. Note that the thickness of the p(g_4_2T-T):CNF nanocomposite films was not changed even after swelling and drying were repeated 3 times over the course of 5 days. Thus, it is clear that CNF remains in the bulk films (see Figure S4a,b). We were unable to carry out tensile deformation experiments with neat p(g_4_2T-T), which became too soft upon swelling. The water uptake of the nanocomposites was estimated by comparing the weight of dry and swollen samples, *w*_dry_ and *w*_swollen_, expressed as the swelling ratio according to:
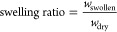
5

as well as the water content in percent (%):
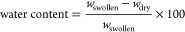
6

All four studied compositions showed similar behavior with a swelling ratio of about 2 and a water content of about 50% (Table S2). Fourier transform infrared spectroscopy (FTIR) spectra of swollen samples featured a broad peak at 3350 cm^–1^, corresponding to the O–H stretch vibration, which significantly decreased in magnitude upon drying under ambient conditions for 15 min (Figure S5). Therefore, tensile deformation of swollen nanocomposites was carried out immediately after their removal from the deionized water bath so that the time in air until the end of the tensile deformation experiment did not exceed 10 min. Tensile deformation experiments revealed that swollen samples displayed a more than one order of magnitude lower Young’s modulus of only *E* = 3–15 MPa, compared to dried samples (Figures 4b, S6a and Table S1). The here observed significant reduction in elastic modulus upon swelling with water is in agreement with previous reports of other types of cellulose nanocomposites, which albeit comprised non-conjugated polymer matrices.^20,50^ To investigate the reversibility of the swelling process, we dried the swollen nanocomposites by placing them in a vacuum oven at 40 °C for ∼24 h. Tensile deformation of swollen and then dried samples indicated that nanocomposites with 11 and 20 vol % CNF were able to regain the same degree of reinforcement, as evidenced by a similar Young’s modulus before and after swelling/drying (Figures 4b and S6b). Instead, for higher contents of 27 and 33 vol % CNF, Young’s moduli of swollen and dried samples were somewhat lower than those of the as-cast material, which we explain with irreversible structural changes upon swelling.

**Figure 5:**
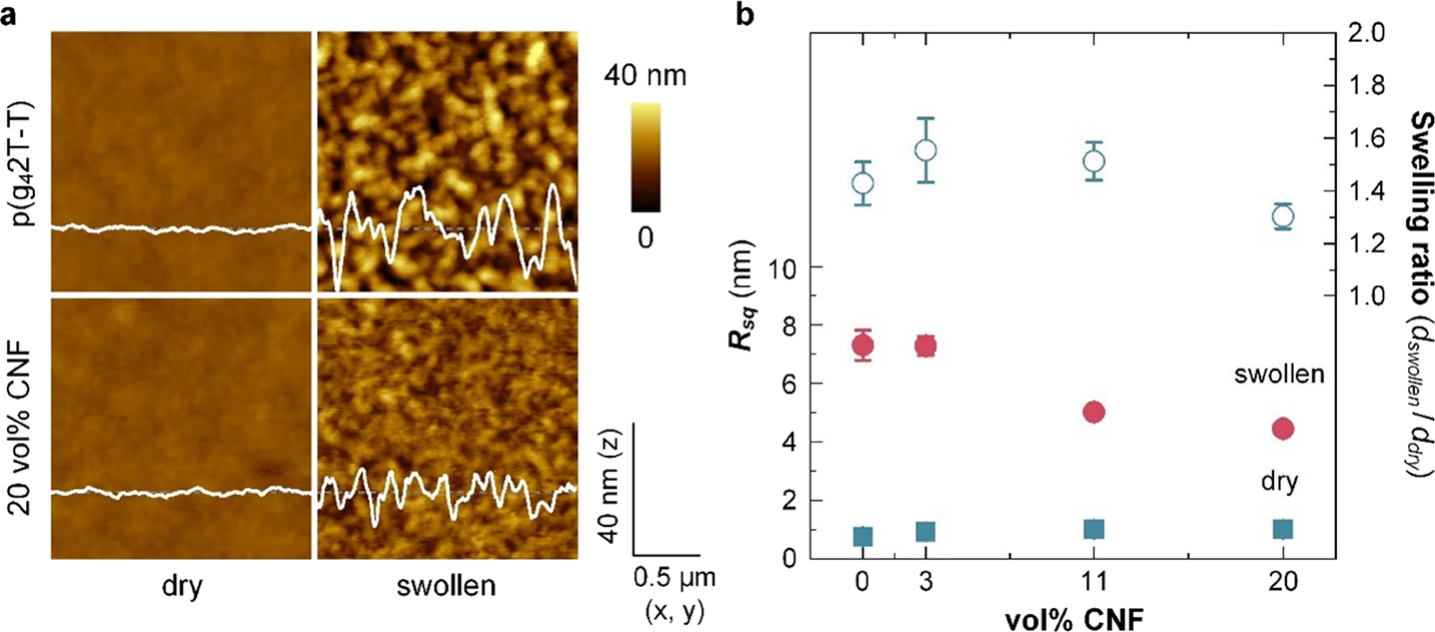
(a) Representative AFM images of neat p(g_4_2T-T) (top) and a nanocomposite film containing 20 vol % CNF (bottom) when dry (left) and swollen, measured while submerged in deionized water (right). Corresponding height profiles are overlayed. Scale-adjusted topography images are depicted in Figure S7. (b) RMS roughness *R*_sq_ (left, solid symbol; mean values and standard error from four measurements) of the dry and submerged films and swelling ratio *d*_swollen_/*d*_dry_ (right, open symbol; mean values and standard error of four measurements) as a function of the CNF content.

AFM allowed us to compare the texture and thickness of dry and swollen thin films. AFM topography images reveal that neat films are smooth with a low root-mean-square (RMS) roughness of *R*_sq_ = 0.8 nm in the case of neat p(g_4_2T-T) and a slightly higher value of *R*_sq_ = 1.0 nm for a nanocomposite film containing 20 vol % CNF (Figures 5 and S6a,c), indicating that CNF is embedded in the film. However, the roughness and thickness significantly increased when submerging the films in deionized water. AFM topography images of films submerged in deionized water revealed a roughness of *R*_sq_ = 7.3 nm and a swelling ratio of *d*_swollen_/*d*_dry_ = 1.43 ± 0.16 for neat, swollen p(g_4_2T-T), and values of 4.4 nm and 1.30 ± 0.09 for a nanocomposite film containing 20 vol % CNF (see Figure 5b; *d*_swollen_ and *d*_dry_ are the thickness of swollen and dry films, respectively). Thin films experience a lesser degree of passive swelling compared to bulk samples (cf. Table S2), which has also been observed for other materials such as ionomers and was explained with confinement effects.^51^ AFM topography images reveal a lower roughness as compared to the neat polymer (Figures 5b and S7b,d). Swollen nanocomposite films feature a lower roughness compared to neat, swollen p(g_4_2T-T), i.e., the presence of CNF results in films with a more fine-grained texture. Repeated swelling and drying were carried out for a nanocomposite film comprising 20 vol % CNF, and an invariant film thickness after five cycles suggests that CNF remains in the films (Figure S4). Hence, we were readily able to fabricate electrochemical devices with the here studied nanocomposites.

**Figure 6:**
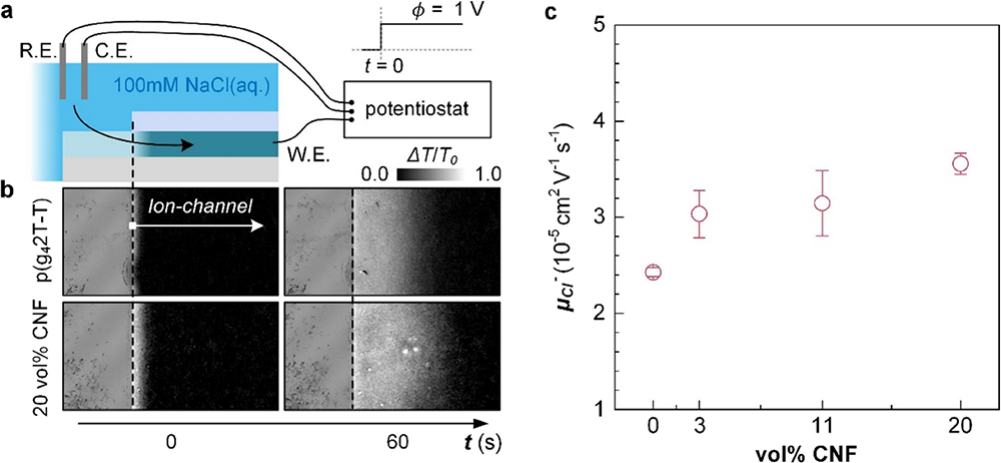
(a) Schematic of planar devices and measurement setup used to extract the mobility of Cl^–^ ions μ_Cl^–^_ (R.E., reference electrode; C.E., counter electrode; and W.E., working electrode). (b) Representative optical images of the channel of devices comprising p(g_4_2T-T) (top) and a nanocomposite comprising 20 vol % CNF (bottom) before applying a potential and after applying a potential ϕ = 1 V for 60 s at the working electrode. The gray scale depicts the relative intensity Δ*I* = *I*_0_ – *I* normalized by the initial intensity *I*_0_. (c) μ_Cl^–^_ as a function of the CNF concentration (mean values and min–max error of two measurements) for an aqueous electrolyte (100 mM NaCl).

The hydrophilic nature of CNFs can be expected to alter the transport of ions into polymer films during electrochemical oxidation, which would influence the operation of electrochemical devices. We constructed planar devices that allowed us to track the moving front of electrochromic color changes upon application of an electrochemical potential, similar to the experiments described by Stavrinidou et al. (see Figure 6a for the schematic of the setup and the Experimental Section for details).^52^ A potential of ϕ = 1 V vs Ag/AgCl was applied at one end of the channel,^53^ and a CCD camera was used to monitor the lateral change in color due to oxidation of the polymer, which requires the ingression of Cl^–^ ions from the aqueous electrolyte (100 mM NaCl) into the film and thus provides information about the mobility of Cl^–^ ions. A comparison of the optical images shown in Figure 6b reveals that after application of the electrical potential for 60 s, the moving front has progressed further into nanocomposite films compared to neat p(g_4_2T-T), indicating a higher ion mobility. The position of the moving front *l*(*t*) as a function of time *t* (see Figure S8a) allowed us to determine the mobility of Cl^–^ ions μ_Cl^–^_ according to (see the Experimental Section for details):
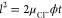
7

For neat p(g_4_2T-T), we extract an ion mobility of μ_Cl^–^_ = 2.4 × 10^–5^ cm^2^ V^–1^ s^–1^ from the plots of *l*^2^ vs *t* (Figure S8b), which increases to 3.6 × 10^–5^ cm^2^ V^–1^ s^–1^ for a nanocomposite film containing 20 vol % CNF (Figure 6c). We conclude that the addition of CNF to p(g_4_2T-T) results in a slightly enhanced mobility of Cl^–^ ions in nanocomposite films.

**Figure 7:**
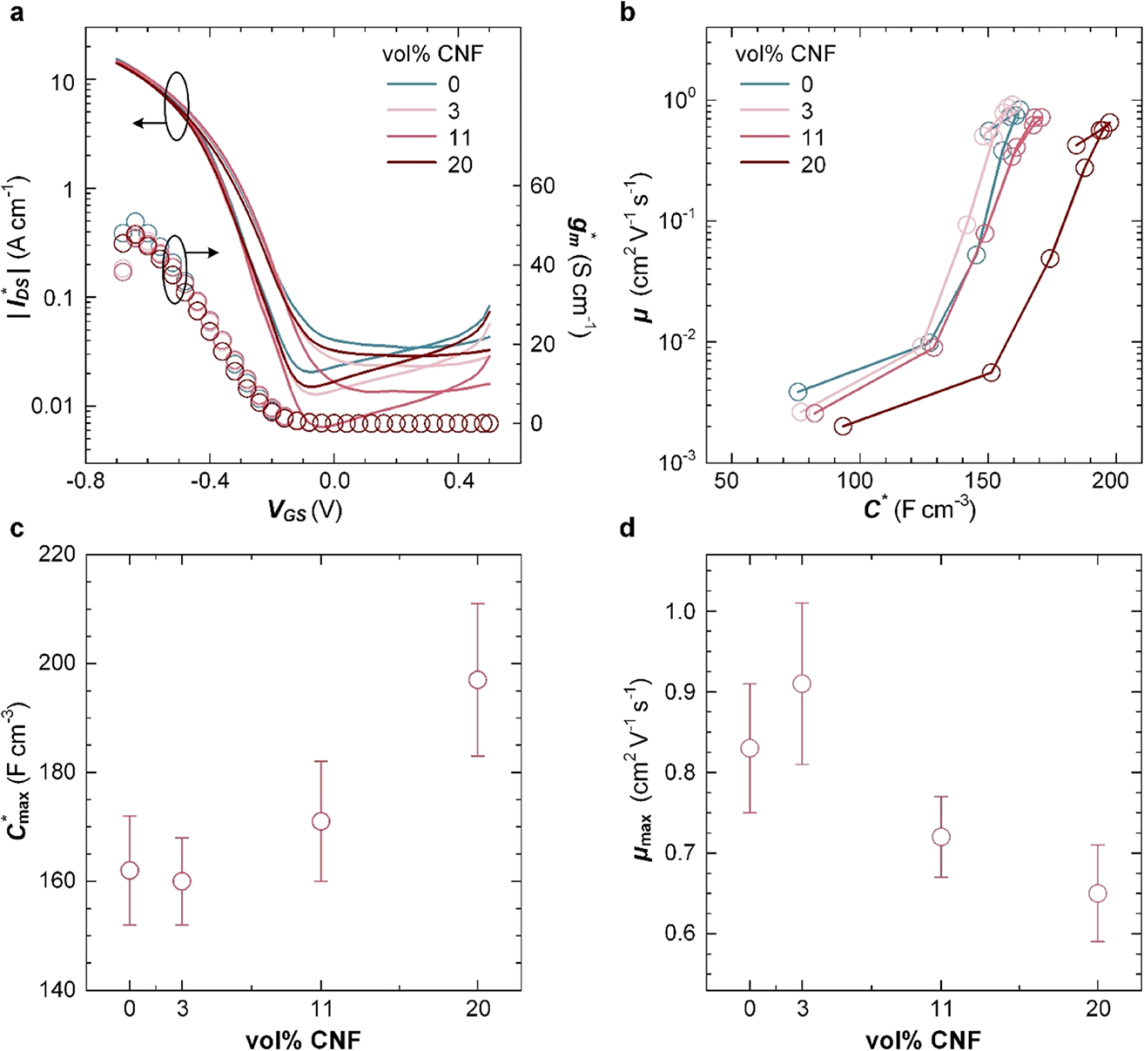
OECTs. (a) Dimension-normalized source–drain current *I*_DS_^*^ = *I*_DS_/(*W* × *d*/*L*) (left) and corresponding transconductance *g*_m_^*^ = *g*_m_/(*W* × *d*/*L*) (right, *V*_DS_ = −0.7 V, *V*_GS_ = +0.5 to −0.7 V), where *W*, *d*, and *L* are the width, thickness, and length of the channel. (b) Trajectory of μ and C* values upon varying *V*_GS_ from +0.1 to −0.6 V; Δ*V* = −0.1 V. (c) Maximum volumetric capacitance *C*_max_^*^ and (d) hole mobility μ_max_ as a function of the CNF concentration (see Table 1 for the description of errors).

OECTs were fabricated to investigate how the addition of CNF impacts the electrochemical response of p(g_4_2T-T). Transfer curves of devices with active layers composed of neat p(g_4_2T-T) were compared with devices based on nanocomposites containing 3, 11, or 20 vol % CNF. All transfer curves feature low current levels at positive gate voltages *V*_GS_ and a sharply increasing dimension-normalized source–drain current *I*_DS_^*^ = *I*_DS_/(*W* × *d*/*L*) below a threshold voltage *V*_th_ ≈ −0.2 V (Figure 7a and Table 1; see Figure S9 for output characteristics; *W*, *d*, and *L* are the width, thickness, and length of the channel), indicating p-type accumulation mode operation. The dimension-normalized transconductance *g*_m_^*^ = |d*I*_DS_^*^/d*V*_GS_| of all devices is comparable (Figure 7a), indicating that the presence of non-conducting CNF does not significantly affect the electrical properties of the materials. The product of volumetric capacitance C* and mobility μ can be obtained from *g*_m_^*^ according to
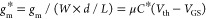
8

**Table 1:** Electrical Properties of Doped Films Comprising Different Amounts of CNF^a^

	electrochemical doping				chemical doping		
vol % CNF	*V*_th_ (V)	μC* (F cm^–1^ V^–1^ s^–1^)	*C*_max_^*^ (F cm^–3^)	μ_max_ (cm^2^ V^–1^ s^–1^)	σ (S cm^–1^)	*N*_p_ (10^26^ m^–3^)	μ_F_4_TCNQ_ (cm^2^ V^–1^ s^–1^)
0	–0.20	135 ± 9	162 ± 10	0.83 ± 0.08	24.7 ± 0.3	0.7 ± 0.2	2.1 ± 0.6
3	–0.18	146 ± 15	160 ± 8	0.91 ± 0.10	24.8 ± 3.2	0.8 ± 0.2	2.1 ± 0.7
11	–0.17	123 ± 5	171 ± 11	0.72 ± 0.05	17.9 ± 0.1	1.0 ± 0.3	1.1 ± 0.3
20	–0.20	130 ± 8	197 ± 14	0.65 ± 0.06	19.5 ± 1.4	0.9 ± 0.3	1.4 ± 0.4

a Electrochemical doping with OECTs (channel width *W* = 200 μm and channel length *L* = 20 μm) and EIS devices: threshold voltage *V*_th_, the product of hole mobility and volumetric capacitance μC* from OECT transfer curves (mean and standard error of values extracted from 8–10 devices), maximum dimension-normalized volumetric capacitance *C*_max_^*^ from EIS (mean and standard error of values extracted from 4 devices), and maximum hole mobility μ_max_. Chemical doping with F_4_TCNQ (20 mol % relative to the g_4_2T-T repeat unit): electrical conductivity σ (mean and min–max error of three measurements of the same sample), polaron density *N*_p_ from analysis of UV–vis spectra (estimated error 30%), and charge-carrier mobility μ_F_4_TCNQ_.

We obtain a value of μC* = 135 F cm^–1^ V^–1^ s^–1^ for neat p(g_4_2T-T), which is not significantly affected by the addition of CNF and comparable to values reported for p(g_3_2T-T), a similar polymer with shorter triethylene glycol side chains.^54^

Electrochemical impedance spectroscopy (EIS) for potentials ranging from −0.4 to 0.8 V vs Ag/AgCl (Figure S10) allowed us to determine the volumetric capacitance C*, which displayed a maximum value *C*_max_^*^ at a potential of +0.4 V vs Ag/AgCl (Figure S11). The C* values from EIS in combination with the μC* values from OECT transfer curves were used to calculate the mobility. Trajectories of μ vs C* reveal a comparable trend for both neat p(g_4_2T-T) and the investigated nanocomposites with 3, 11, and 20 vol % CNF (Figure 7b). Both parameters first increase and reach maximum values at *V*_GS_ = −0.4 V, followed by a slight decrease at higher oxidation potentials, in agreement with previous reports for similar polymers such as a thienothiophene–thiophene copolymer with oligoether side chains.^54^ The maximum capacitance *C*_max_^*^ increased with the CNF content from 162 to 197 F cm^–3^ (Figure 7c and Table 1). Instead, the maximum mobility μ_max_ slightly decreased with the CNF content from μ_max_ = 0.83 cm^2^ V^–1^ s^–1^ for neat p(g_4_2T-T) to 0.65 cm^2^ V^–1^ s^–1^ for the nanocomposite with 20 vol % CNF (Table 1 and Figure 7d). The increase in *C*_max_^*^ indicates that the presence of CNF aids the accumulation of charge carriers upon application of a negative *V*_GS_, which compensates for the slight decrease in charge-carrier mobility, overall resulting in invariant values of μC* and hence a transconductance that is not affected by the presence of CNF (see Figure 7a). We argue that the more fine-grained texture of swollen nanocomposite films as compared to neat p(g_4_2T-T) (see the AFM images in Figure 5) results in additional grain boundaries that slightly impede charge transport but increase the internal surface area where electrochemical oxidation can occur.

**Figure 8:**
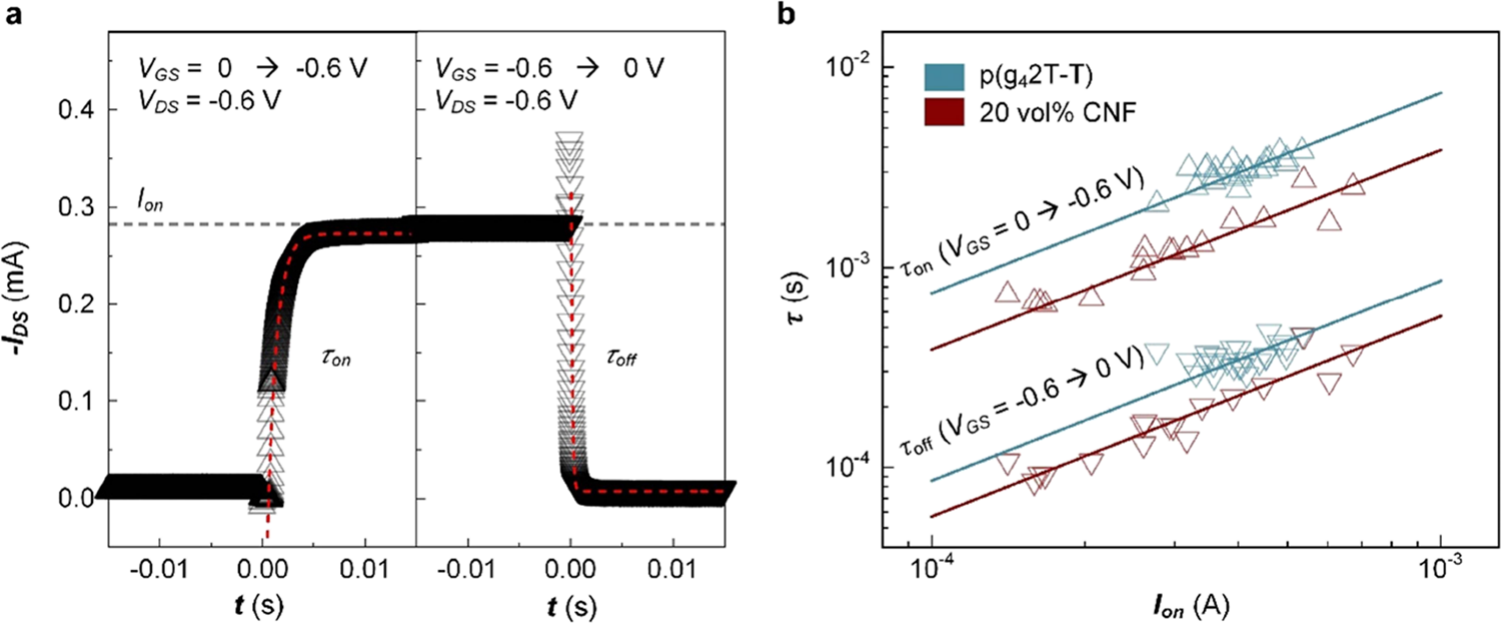
Transient analysis of OECT devices. (a) Representative plot of the *I*_DS_ response (triangles: data; dashed lines: exponential fits using eqs 9 and 10) upon application of voltage steps at the gate electrode (left: *V*_GS_ = 0 to −0.6 V, right: *V*_GS_ = −0.6 to 0 V). (b) Plot of the time constants τ_on_ (upper triangles) and τ_off_ (lower triangles) vs *I*_DS_ as well as linear fits (solid lines) for devices based on neat p(g_4_2T-T) (blue) and nanocomposites comprising 20 vol % CNF (red).

The addition of CNF increases the ionic mobility without affecting μC*, which may accelerate the response of OECTs to changes in the input gate potential, an advantage for circuit design. To verify that CNF composites display faster switching behavior, we fabricated OECT devices with a patterned active layer with a size of 220 × 100 μm^2^ for the channel (*W*/*L* = 100 μm/20 μm). The contact region had a size of 100 × 100 μm^2^ to minimize parasitic capacitance, which can strongly influence the transient response. Then, the drain current *I*_DS_ was recorded while applying a constant *V*_DS_ of −0.6 V and voltage steps on the gate electrode, i.e., *V*_GS_ = 0 to −0.6 V and −0.6 to 0 V for turning devices ON and OFF, respectively. Time constant values τ_on_ and τ_off_ were extracted from the time-dependent drain current by using eqs 9 and 10:
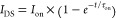
9
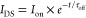
10where *I*_on_ is a steady-state on-current at *V*_GS_ = −0.6 V and *V*_DS_ = −0.6 V, and *t* is the time after applying the voltage step (see Figure 8a). To facilitate a precise analysis of the transient response, we obtained τ and *I*_on_ values from 20 devices, which were prepared with an identical mask layout. The transient response of OECTs depends on the material properties (e.g., C* and μ_Cl^–^_) as well as the exact device dimensions (e.g., channel dimensions and area of the active layer) and parasitic capacitance from the metal electrodes. However, since both *I*_on_ and τ are proportional to the thickness of the active layer, plots of τ vs *I*_on_ facilitate a fair comparison of the transient response of different active-layer materials (Figure 8b).

OECTs based on p(g_4_2T-T):CNF nanocomposites displayed a higher switching speed than devices with a neat p(g_4_2T-T) active layer. For *I*_on_ = 0.5 mA, for example, we extrapolate values of τ_on_ = 3.72 ms and τ_off_ = 0.43 ms for neat polymer devices, while for nanocomposite devices, we obtain lower values of τ_on_ = 1.93 ms and τ_off_ = 0.29 ms (Figure 8). Hence, the addition of 20 vol % CNF to p(g_4_2T-T) results in a 1.9 and 1.5 times faster ON and OFF switching speed, respectively. Even though the nanocomposite has a slightly higher volumetric capacitance and lower charge-carrier mobility than the neat polymer, which would suggest lower switching speeds for identical current output, the higher ionic mobility of the nanocomposite films appears to improve the transient response of OECTs.

To rule out that differences in active swelling, i.e., water uptake upon the application of an electrochemical potential, cause the observed decrease in μ_max_ with the CNF content, we also carried out chemical doping with 2,3,5,6-tetrafluoro-tetracyanoquinodimethane (F_4_TCNQ). P(g_4_2T-T) and CNF were coprocessed with 20 mol % F_4_TCNQ (relative to the g_4_2T-T repeat unit), resulting in thin films with an electrical conductivity of about 20 S cm^–1^ (Table 1). Previous studies have shown that the selected dopant concentration results in highly doped films and does not negatively affect the nanostructure of the polymer.^6,12^ UV–vis spectra of doped films featured distinct polaronic absorption peaks in the near-infrared part of the spectrum as well as two additional peaks around 1.5 eV that are characteristic of F_4_TCNQ anions. We estimated the fraction of ionized dopant molecules by comparing the UV–vis spectra around 3 eV with previously reported absorption spectra of neutral F_4_TCNQ and its anion (Figure S12).^55^ Assuming that each F_4_TCNQ anion gives rise to one polaron on the polymer backbone, we were able to estimate the number of charge carriers (polarons) *N*_p_, which allowed us to determine the charge-carrier mobility according to
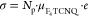
11where *e* is the elementary charge (Table 1). A value of *N*_p_ ≈ 0.7 × 10^26^ m^–3^ yields a value of μ_F_4_TCNQ_ = 2.1 cm^2^ V^–1^ s^–1^ for neat p(g_4_2T-T), which decreases to 1.4 cm^2^ V^–1^ s^–1^ for the nanocomposite with 20 vol % CNF, in agreement with the slight decrease in charge-carrier mobility deduced from OECT devices (Table 1).

## Conclusions

Coprocessing of the polar polythiophene p(g_4_2T-T) with carboxymethylated CNF allows the preparation of nanocomposites that in the dry state feature a Young’s modulus of more than 400 MPa, an increase by more than two orders of magnitude compared to the neat polymer. Moreover, passive swelling of the nanocomposites resulted in a reversible decrease in modulus to 10 MPa or less. The addition of CNF to p(g_4_2T-T) enhances the mobility of Cl^–^ ions but results in a slight decrease in electronic mobility compared to the neat polymer, with values of 3.6 × 10^–5^ and 0.65 cm^2^ V^–1^ s^–1^ for nanocomposites comprising 20 vol % CNF. At the same time, nanocomposites featured an improvement in volumetric capacitance, reaching a value of *C*_max_^*^ = 197 F cm^–3^ for nanocomposites comprising 20 vol % CNF. Overall, OECTs reveal an invariant transconductance but a higher switching speed upon the addition of CNF to the channel. Evidently, CNF can be used as a reinforcing agent for polymers with oligoether side chains, which allows one to modulate the mechanical properties and, in addition, improves the electrochemical performance of the polymer. The combination of reversible passive swelling and invariant transconductance facilitates the design of mechanically adaptive mixed ionic-electronic conductors for wearable electronics and bioelectronics.

## Experimental Section

### Materials

p(g_4_2T-T) with a number-average molecular weight *M*_n_ = 21 kg mol^–1^ and polydispersity index PDI = 5.9 was synthesized according to a previously reported procedure.^6^ A 2.6 wt % aqueous dispersion of carboxymethylated CNF with a degree of substitution of 0.1 was provided by the Research Institutes of Sweden (RISE). Solvent exchange from water to DMF resulted in a dispersion of 0.9 wt % CNF in DMF, using the following procedure: (1) 400 mL of DMF (purity ≥99.9% purchased from Sigma-Aldrich) was added to a round-bottom flask containing 2 g of the aqueous CNF dispersion; (2) the mixture was sonicated for 20 min and then stirred for 30 min using a homogenizer; and (3) a rotary evaporator at a temperature of 80 °C was used to remove the solvent. The above process was repeated 4 times to ensure complete water removal and exchange to DMF. F_4_TCNQ and silver paint were purchased from TCI Chemicals and Agar Scientific, respectively.

### Sample Preparation

Nanocomposites were prepared by stirring a solution of p(g_4_2T-T) in DMF (8 g L^–1^ for thin films or 12 g L^–1^ for thick films) with a dispersion of 0.9 wt % CNF in DMF at different ratios for 1 h using a magnetic stirrer, resulting in p(g_4_2T-T):CNF dispersions that contained a solid content of 0.6 to 33 vol % CNF [calculated assuming a density of 1 and 1.6 g cm^–3^ for p(g_4_2T-T) and CNF]. Doping with F_4_TCNQ was done by coprocessing p(g_4_2T-T):CNF mixtures in DMF with F_4_TCNQ (2 g L^–1^ in acetonitrile). Thin films for spectroscopy and conductivity measurements (thickness *d* ≈ 70 nm) as well as for electrochemical characterization (thickness *d* = 140–230 nm for OECTs and liquid AFM and 50 nm for EIS devices) were prepared by wire bar coating on glass substrates at 45 °C using a K101 control coater from RK Print Instruments (wire diameter = 50 μm). Thick films for tensile testing and WAXS were fabricated by drop casting multiple layers on glass substrates at 45 °C (thickness *d* ≈ 100 μm), followed by drying under vacuum for ∼48 h and finally being removed from the glass substrate with a sharp blade. The film thickness of thin and thick films was measured with AFM or an AlphaStep D-100 profilometer from KLA and microcalipers, respectively.

### Atomic Force Microscopy (AFM)

AFM images were recorded in tapping mode using a MultiMode SPM microscope equipped with a Nanoscope IV Controller from Veeco (Figure 2) or an NTEGRA NT-DMT instrument (Figures 6 and S7). Topography images of swollen films were obtained in liquid AFM mode in deionized water after immersing the as-prepared films in deionized water for 12 h.

### Wide-Angle X-ray Scattering (WAXS)

Transmission WAXS diffractograms were obtained under vacuum using a Mat:Nordic instrument from SAXLAB equipped with a Rigaku 003+ high brilliance microfocus Cu-radiation source (wavelength = 1.5406 Å) and a Dectris Pilatus 300K detector. Samples were placed at a distance of 134 mm from the sample and irradiated for 1800 s. The sample-to-detector distance was calibrated using silver behenate powder. The SaxsGUI software was used for data reduction.

### Tensile Testing

Tensile testing of free-standing thick films was performed using a Q800 dynamic mechanical analyzer from TA Instruments at room temperature in controlled force mode with a force rate of 0.005 N/min using a gauge length of 3.8 mm. To ease fastening between the linear film tension clamps, samples of neat p(g_4_2T-T) and nanocomposites containing up to 4 vol % CNF were fixated in a paper frame that was cut prior to tensile testing.

### UV–vis–NIR Absorption Spectroscopy

UV–vis–NIR spectra of thin films were recorded with a PerkinElmer Lambda 1050 spectrophotometer.

### Fourier Transform Infrared Spectroscopy (FTIR)

FTIR spectra were recorded in attenuated total reflectance (ATR) mode using a PerkinElmer Frontier FTIR instrument equipped with a GladiATR attachment from Pike Technologies.

### Ion Mobility Measurements

Active layers were bar-coated on pre-cleaned indium-doped tin oxide (ITO)-coated glass substrates and patterned with a swab soaked in chloroform (width = 10 mm; length = 30 mm). Then, paraffin films were deposited on the ion-channel region and molten by placing the devices on a hot plate at 60 °C for 5 min. During the measurements, one end of the devices was electrically connected with a Cu clip, and the other end was dipped in an aqueous electrolyte (100 mM NaCl). A potential of ϕ = 1 V vs Ag/AgCl was applied at the ITO working electrode with a Keithley 2400 source-measure unit using a three-electrode configuration that comprised a Ag/AgCl reference electrode and a Pt counter electrode. The potential of ϕ = 1 V vs Ag/AgCl was applied for 5 min, and a video of the channel was recorded with a Canon EOS RP camera. To remove the background, all images (frame rate = 30 fps) of the video were divided by a frame recorded at 0 V vs Ag/AgCl (dedoped state). Intensity profiles along the ion channels were acquired for each frame using the ImageJ software. For each normalized intensity profile, the distance *l* between the start of the channel and the location where the intensity had dropped to 50% of the initial value was determined using MATLAB. The ion mobility was extracted by plotting *l*^2^ vs *t* (Figure S8b; see eq 7).

### OECT Fabrication and Characterization

The metal electrodes of OECT devices were prepared via lithography. First, the source and drain electrodes were defined via a lift-off process using a Karl Suss MA6 contact aligner and a Kurt J Lesker PVD225 e-beam evaporator on cleaned Marienfeld soda lime glass slides, resulting in channels with a width *W* = 200 μm and length *L* = 20 μm. Then, active layers with a thickness of *d* = 140–230 nm were wire bar-coated. The active layers are not patterned but partially removed near the contact pads with a swab soaked in chloroform. Finally, a 20–30 μL drop of an aqueous electrolyte (100 mM NaCl) was placed on top of the active layer. Device characterization was conducted with two MATLAB-controlled Keithley 2400 source-measure units, with a Ag/AgCl reference electrode (RE-1B from ALS) immersed in the electrolyte as the gate electrode. For analysis of the transient response, the drain current was electrically bypassed and recorded using a Stanford Research Systems SR570 preamplifier and a DAQ board from National Instruments.

### Electrochemical Impedance Spectroscopy (EIS)

Films with a thickness of *d* ≈ 50 nm were wire bar-coated on ITO-coated glass substrates (Ossila, 20 Ω sq^–1^). The active layer was patterned with a swab soaked in chloroform, and its area (*A* ≈ 40 mm^2^) and thickness were measured with a CCD camera and AFM, respectively. Finally, the ITO contact was contacted with crocodile clips, passivated with paraffin wax, and then, the sample was immersed in an aqueous electrolyte (100 mM NaCl). EIS spectra were obtained with a CH instrument CHI 650D using a conventional three-electrode configuration that comprised a Ag/AgCl reference electrode and a Pt counter electrode. The offset potential was varied from +0.4 to −0.8 V vs Ag/AgCl with a 20 mV peak-to-peak sinusoidal signal (frequency of 10^–1^ to 10^5^ Hz). The electrochemical capacitance of the active layer was extracted from EIS data using the EIS Spectrum Analyser software and an equivalent circuit model *R*_e_[*R*_s_*C*_s_[*R*_c_[*R*_a_*C*_a_]]], where *R*_e_, *R*_s_, *C*_s_, *R*_c_, *R*_a_, and *C*_a_ are the resistance of the electrolyte, electrochemical resistance/capacitance of the ITO substrate, contact resistance between the substrate and the active layer, and electrochemical resistance/capacitance of the active layer (see Figure S10a for a circuit diagram). The obtained values for *C*_a_ were normalized by the volume (=*d* × *A*) of each sample.

### Electrical Characterization

The sheet resistance *R*_s_ = π/ ln 2 · *V*/*I* of thin films on glass substrates, where *V* and *I* are the measured voltage and current, respectively, was measured with a four-point probe setup from Jandel Engineering (cylindrical probe head, RM3000) using co-linear tungsten carbide electrodes with an equidistant spacing of 1 mm. The electrical conductivity was calculated according to σ = 1/(*dR*_s_), where *d* is the film thickness.

## Supporting Information Available

DMA thermograms, WAXS diffractograms, tensile deformation stress–strain curves and Young’s moduli, swelling ratios, FTIR spectra, AFM images, analysis of moving front experiments, OECT output characteristics, EIS spectra, volumetric capacitance, and UV–vis spectra (PDF)
