# Supplementary material for: Mechanically Adaptive Mixed Ionic-Electronic Conductors Based on a Polar Polythiophene Reinforced with Cellulose Nanofibrils
Source: ACS Appl Mater Interfaces. 2023 Jun 1;15(23):28300–9. doi: 10.1021/acsami.3c03962 (PMC10273225; doi:10.1021/acsami.3c03962)
Supplement: Supplementary file 1 — am3c03962_si_001.pdf [file am3c03962_si_001.pdf]

Supporting Information

Mechanically Adaptive Mixed Ionic-Electronic Conductors based on a Polar Polythiophene Reinforced with Cellulose Nanofibrils

Mariza Mone,[1,2 †] Youngseok Kim,[1†] Sozan Darabi,[1,2] Sepideh Zokaei,[1] Lovisa Karlsson,[1] Mariavittoria Craighero,[1] Simone Fabiano,[3,4] Renee Kroon,[3,4] Christian Müller[1,2]*

[1]Department of Chemistry and Chemical Engineering, Chalmers University of Technology, 412 96 Göteborg, Sweden

[2]Wallenberg Wood Science Center, Chalmers University of Technology, 412 96 Göteborg, Sweden

[3]Laboratory of Organic Electronics, Department of Science and Technology, Linköping University, 602 21 Norrköping, Sweden

[4]Wallenberg Wood Science Center, Linköping University, 602 21 Norrköping, Sweden.

[†]These two authors contributed equally.

*christian.muller@chalmers.se

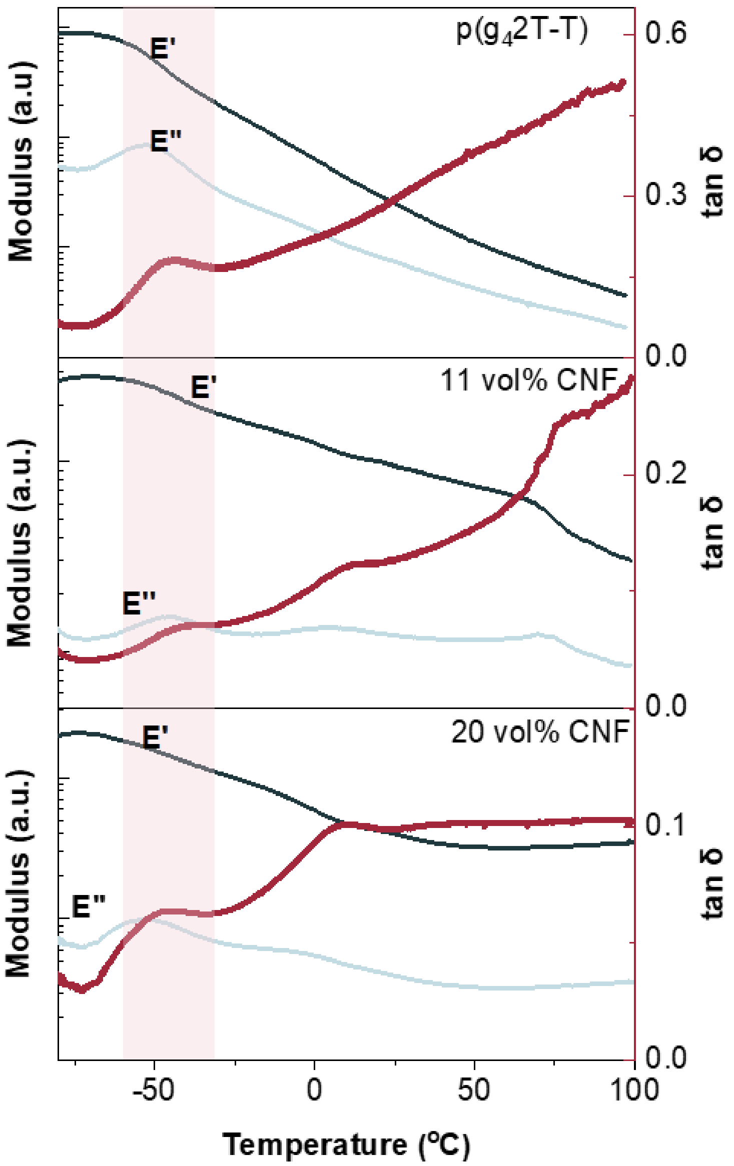

Figure S1. Storage and loss modulus, E' and E'', and loss tangent $\tan\delta = E''/E'$ of neat p($g_4$2T-T) (top) and p(g42T-T):CNF nanocomposites containing 11 vol% CNF (middle) and 20 vol% CNF (bottom).

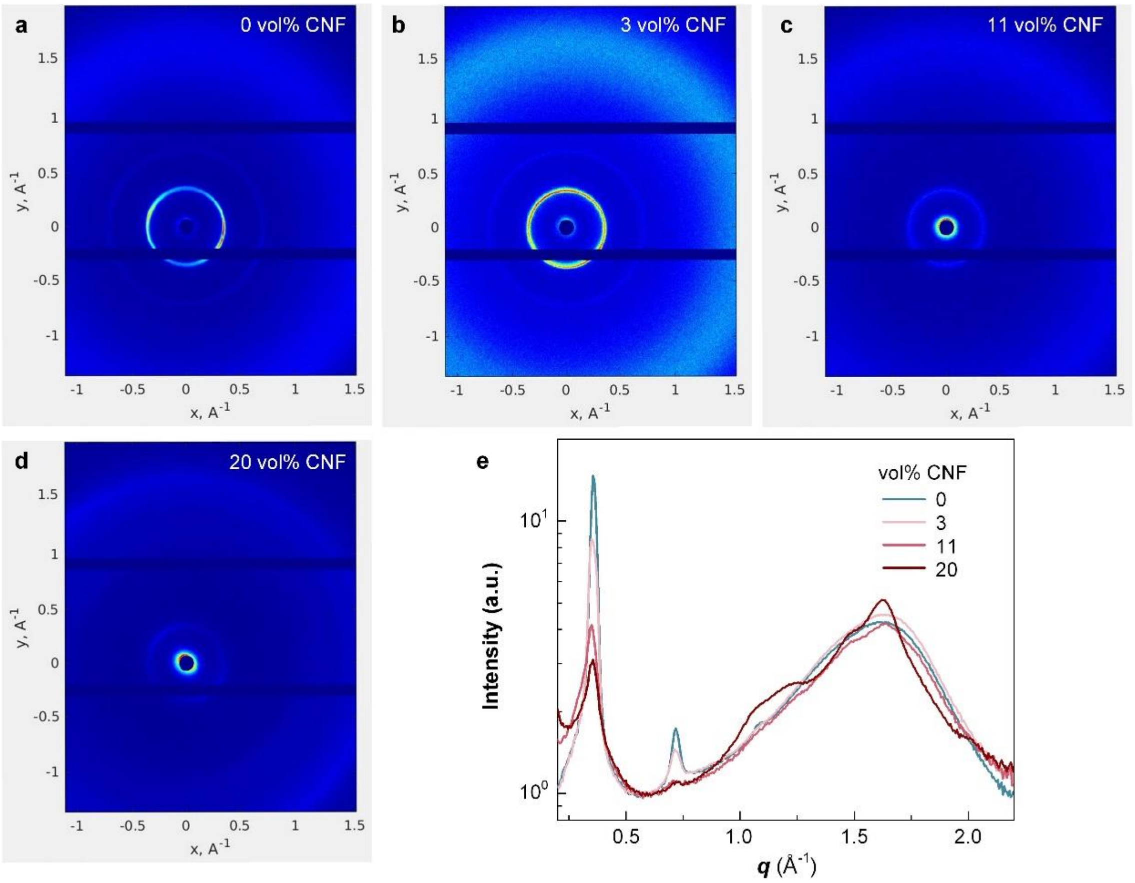

Figure S2. 2D WAXS patterns of thick p(g$_4$2T-T) and nanocomposite films containing (a) 0, (b) 3, (c) 11, and (d) 20 vol% CNF. (e) Radial integration of 2D WAXS data of thick films. The vol% CNF for the nanocomposites is indicated in the legend.

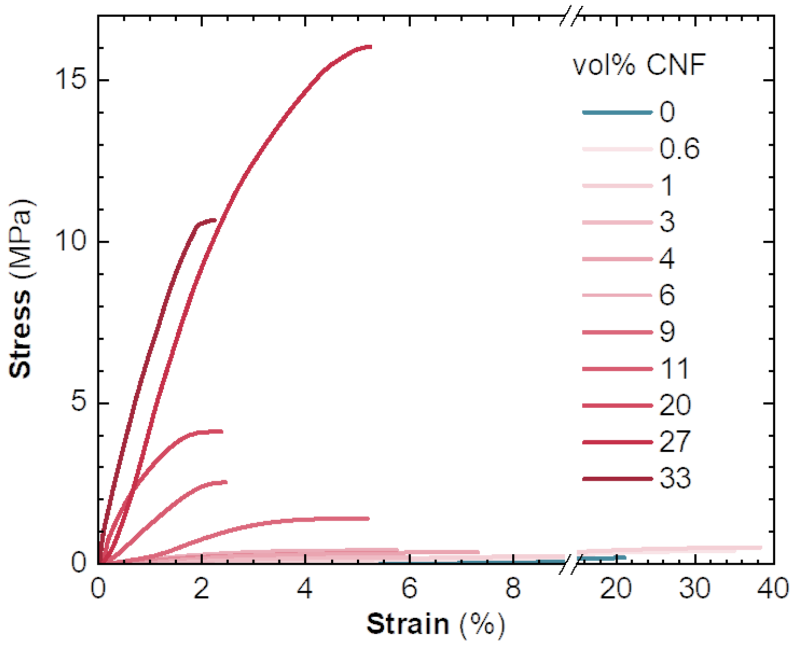

Figure S3. Stress–strain curves recorded at room temperature by tensile deformation of samples of neat p(g$_4$2T-T) (dark blue) and the polymer reinforced with CNF; vol% CNF as indicated by the legend.

Table S1. Young's modulus of dry p(g$_4$2T-T):CNF nanocomposites E_{dry}, of samples swollen with deionized water $E_{swollen}$, and of samples that had been swollen and dried again E'_{dry}.

vol% CNF	E_{dry} (MPa)	$E_{swollen}$ (MPa)	E'_{dry} (MPa)
0	1.2 ± 0.1		
0.6	2.6 ± 0.8		
1.2	2.5		
3	5.8 ± 0.6		
4	8.8 ± 1.5		
6	15 ± 5		
9	50 ± 17		
11	105 ± 33	4 ± 1	87 ± 26
20	421 ± 170	15	372 ± 60
27	587 ± 33	3.0 ± 0.2	126 ± 41
33	634 ± 140	3.0 ± 0.1	274 ± 71

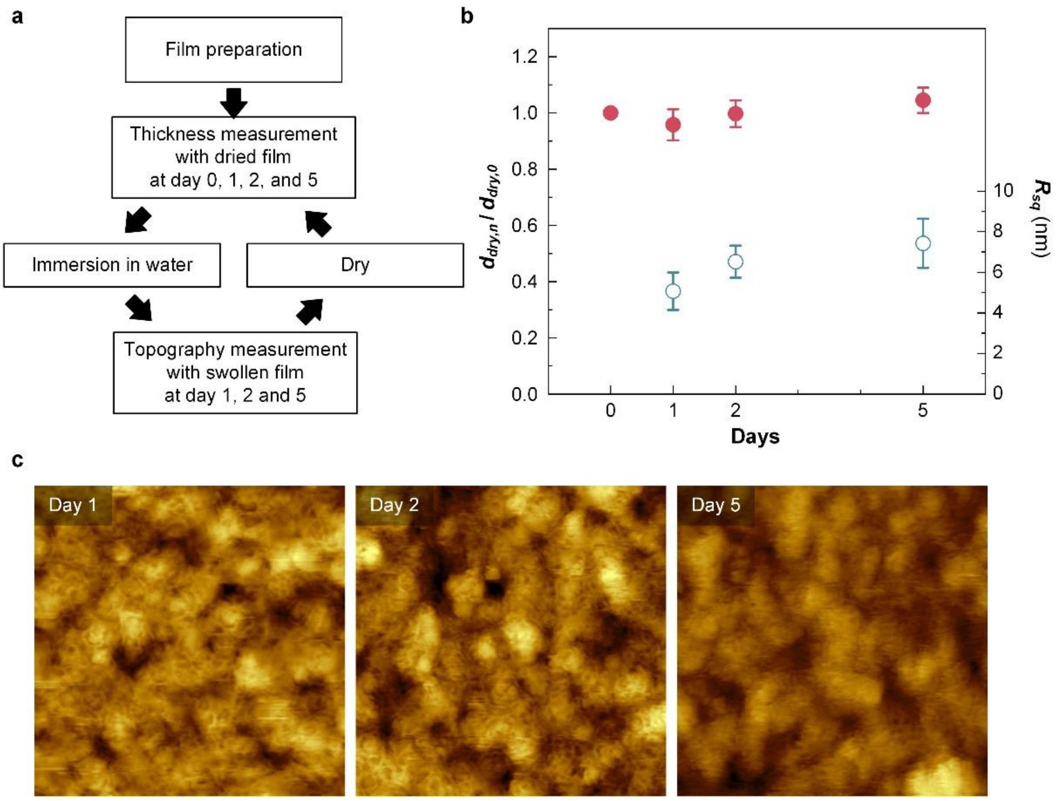

Figure S4. AFM analysis of films that experienced repeated swelling/drying. (a) Flowchart of the experiment. The nanocomposite films (20 vol% CNF) were once per day immersed in deionized water and dried again, and the roughness of swollen films as well as the thickness of dried films were measured. (b) Plots of relative thickness change (left, $d_{dry,n}$ / $d_{dry,0}$, where $d_{dry,n}$ and $d_{dry,0}$ are the thickness on the n-th day and the thickness measured immediately after film preparation, respectively), and roughness (right) of the swollen film as a function of time. (c) Representative AFM images on day 1, 2 and 5.

Table S2. Swelling ratio and water content of nanocomposites swollen with deionized water.

vol% CNF	swelling ratio	water content (%)
11	1.97 ± 0.13	49 ± 3
20	2.38 ± 0.09	58 ± 2
27	2.04 ± 0.11	51 ± 3
33	2.07 ± 0.22	52 ± 5

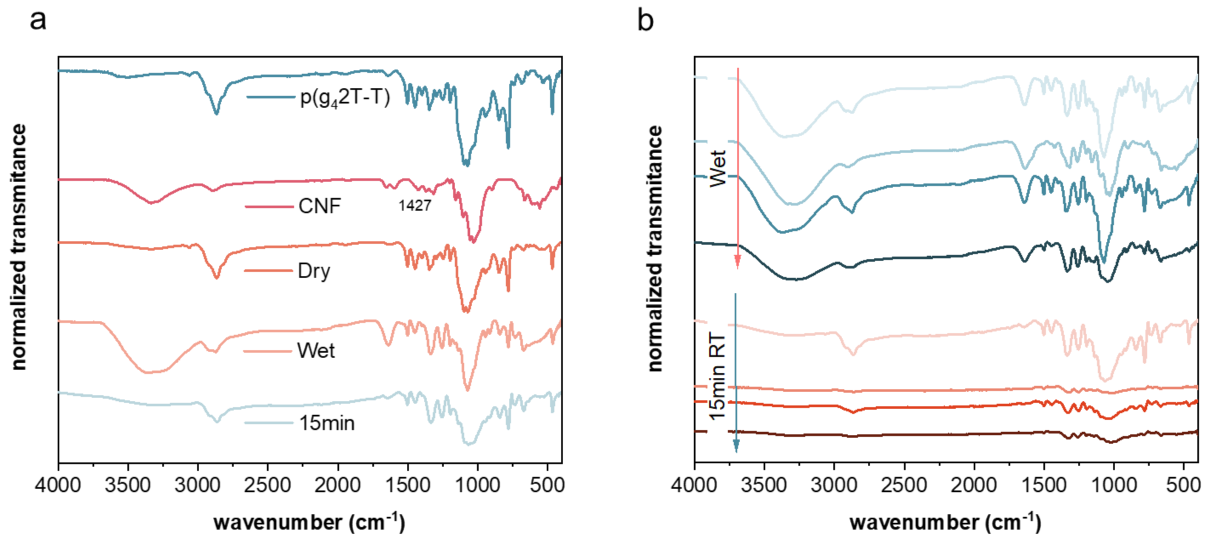

Figure S5. (a) FTIR transmittance spectra of p($g_4$2T-T), CNF, and the nanocomposite containing 11 vol% of CNF that was initially dry (dry), swollen in deionized water (wet), and finally left at ambient conditions for 15min (15 min). (b) FTIR transmittance spectra of p($g_4$2T-T):CNF nanocomposites swollen in deionized water (wet) and re-dried at ambient conditions for 15min (15 min RT). The compositions shown correspond to 11, 20, 27, and 33 vol% CNF. The direction of the arrow indicates increasing CNF loading.

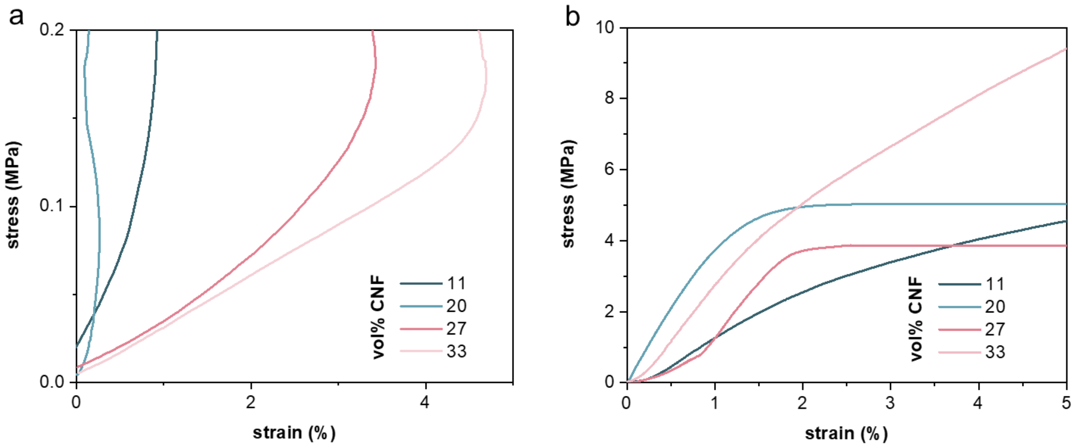

Figure S6. Stress-strain curves recorded by tensile deformation at room temperature of (a) nanocomposites swollen with deionized water (the initial slope was used to determine the Young's modulus; note that the slope increases with time because the samples dry during the experiment); and (b) of nanocomposites that had been swollen and dried.

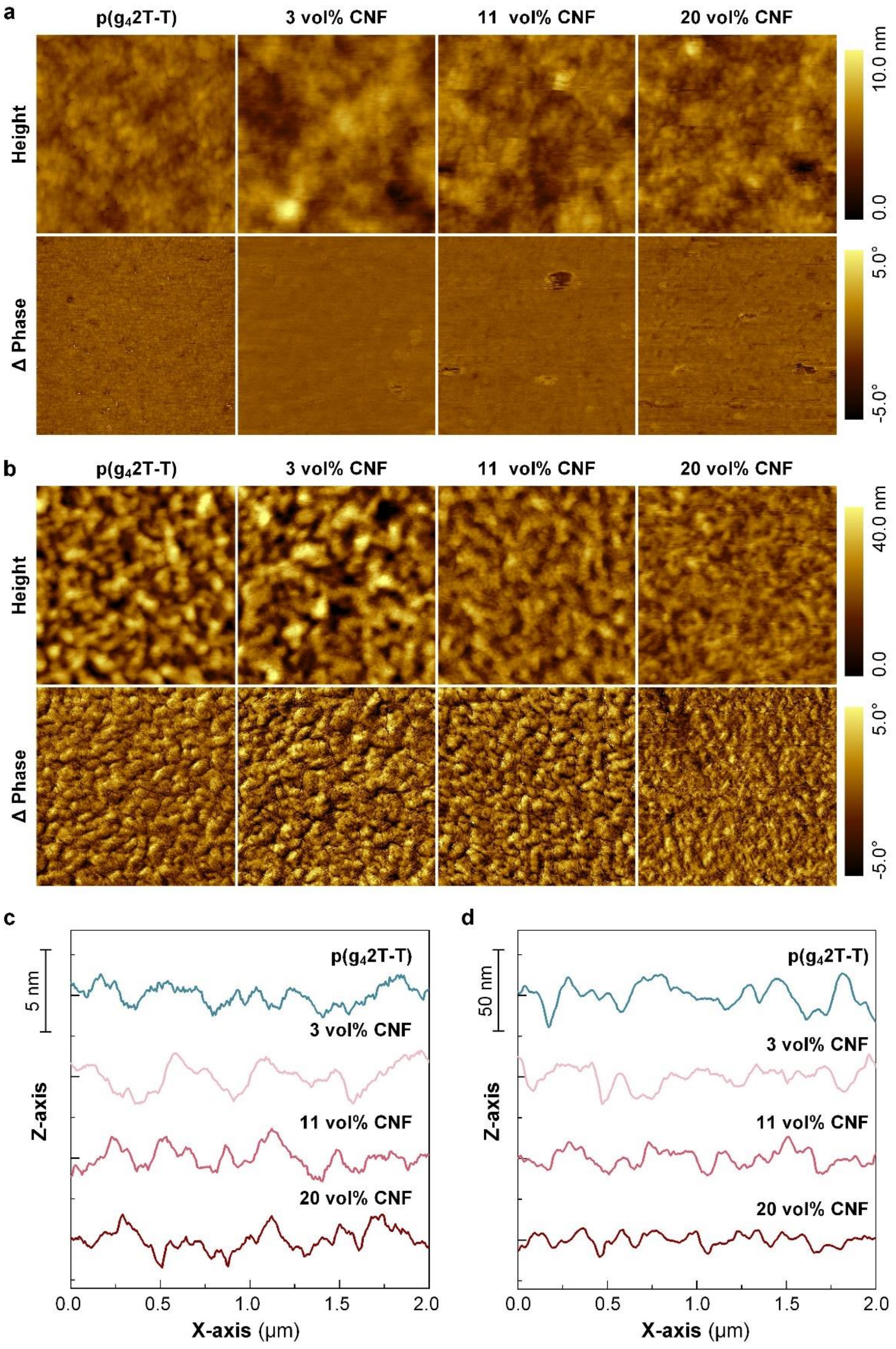

Figure S7. (a)/(b) AFM height (upper) and phase images (lower), and (c)/(d) corresponding line profiles of dry/swollen films.

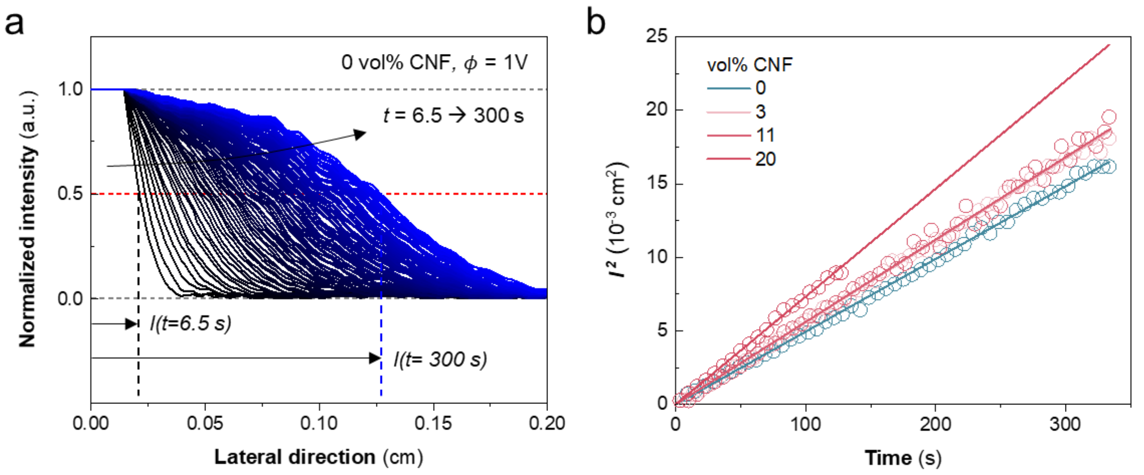

Figure S8. (a) Normalized intensity profiles along the ion channel from t = 6.5 (black) to 300 s (blue) for neat p(g$_4$2T-T) films. (b) A plot of l^2 as a function of time. The ion mobility was extracted from the slope of linear fits (solid lines) of the measured values (open circles).

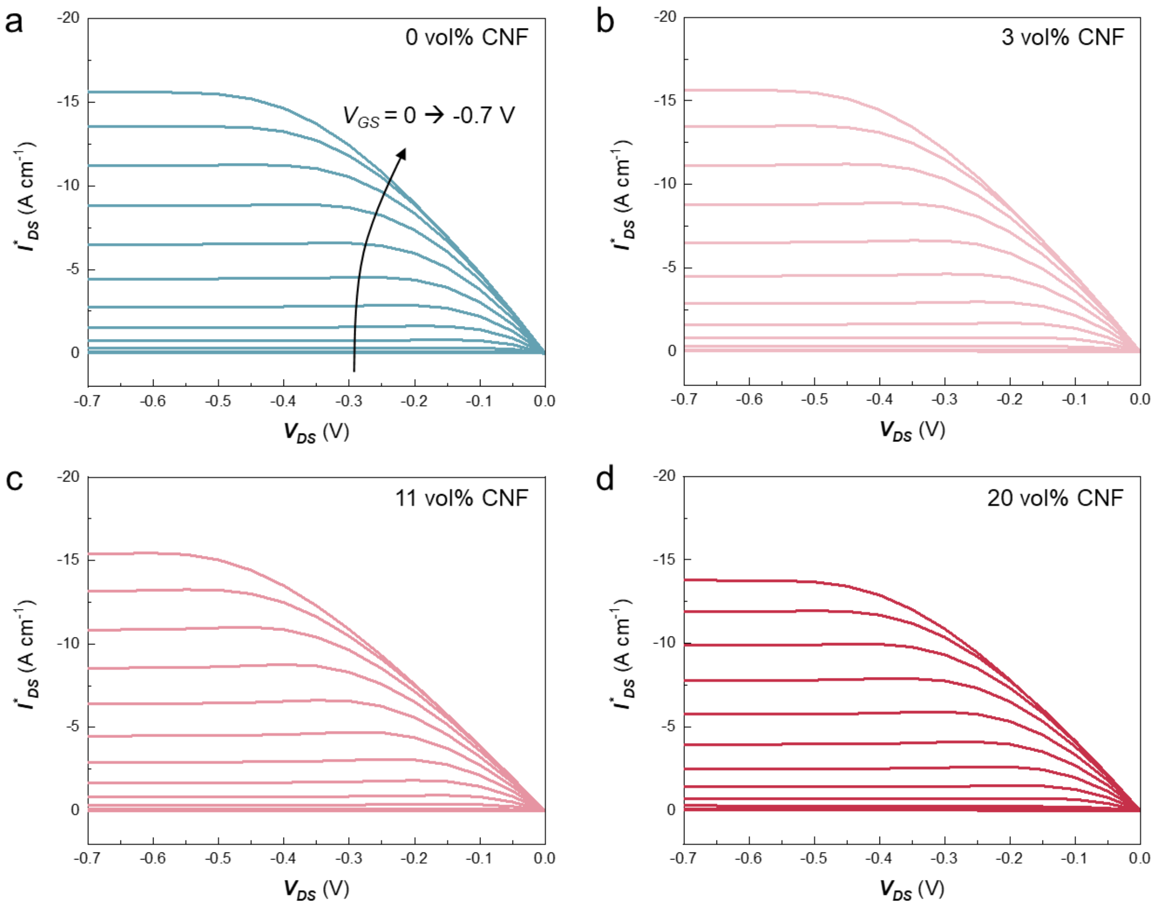

Figure S9. Output characteristic curves (V_{DS}: 0.0 to −0.7 V, V_{GS}: 0.0 to -0.7 V with 0.05 V step) of (a) p(g$_4$2T-T) and p(g$_4$2T-T):CNF nanocomposites with (b) 3 vol% (c) 11 vol% and (d) 20 vol% CNF. The measured drain current I_{DS} was normalized with the channel dimensions of each sample, $I^*_{DS}= I_{DS}/(W \times d/L)$.

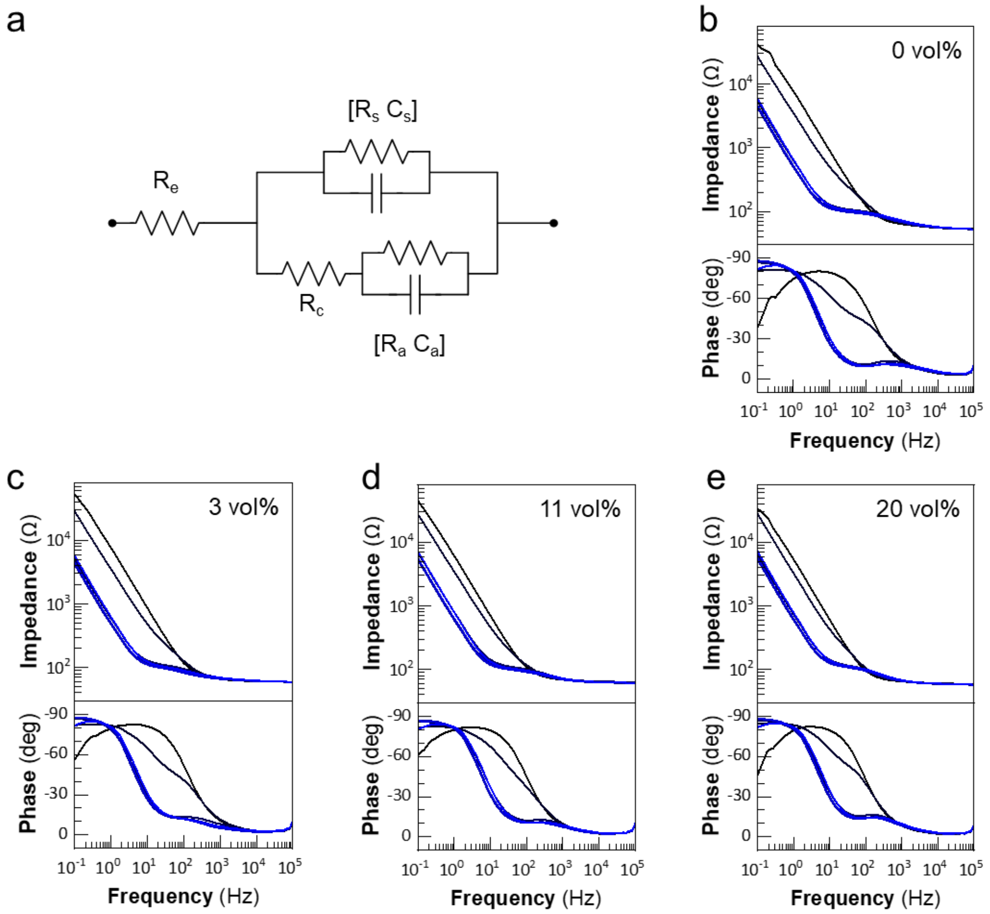

Figure S10. (a) Equivalent circuit model for extraction of the electrochemical capacitance of the active layer. R_e, R_s, C_s, R_c, R_a and C_a are the resistance of the electrolyte, the electrochemical resistance/capacitance of the ITO substrate, the contact resistance between the substrate and active layer, and the electrochemical resistance/capacitance of the active layer, respectively. Electrochemical impedance spectroscopy (EIS) spectra recorded at offset potentials ranging from -0.4 (black) to 0.8 V (blue) with a 0.2 V step of (b) p($g_4$2T-T) and p($g_4$2T-T):CNF nanocomposite with (c) 3 vol%, (d) 11 vol%, and (e) 20 vol% CNF.

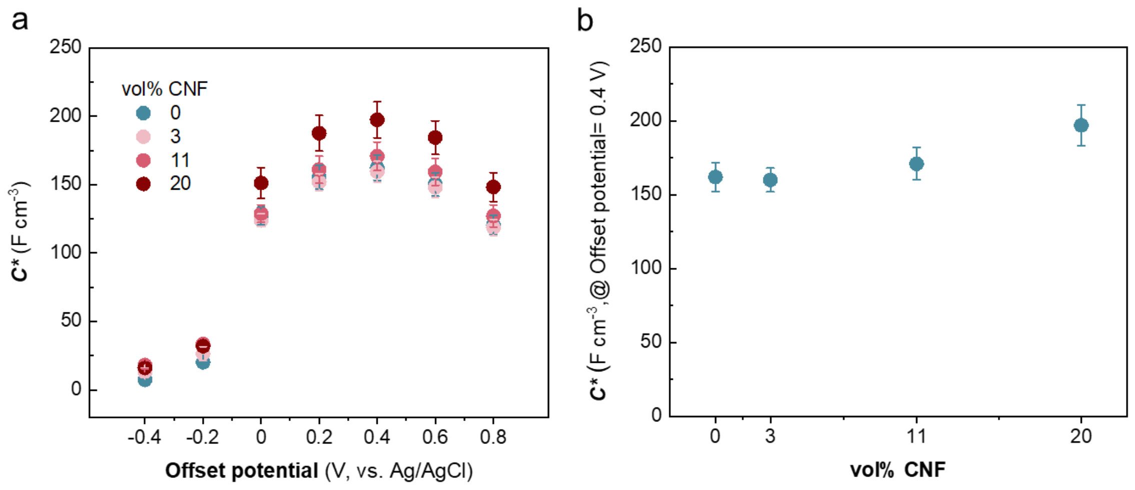

Figure S11. (a) The volumetric capacitance as a function of the offset potential for p(g$_4$2T-T) and p(g$_4$2T-T):CNF nanocomposites, and (b) the maximum volumetric capacitance values as a function of the concentration of CNF.

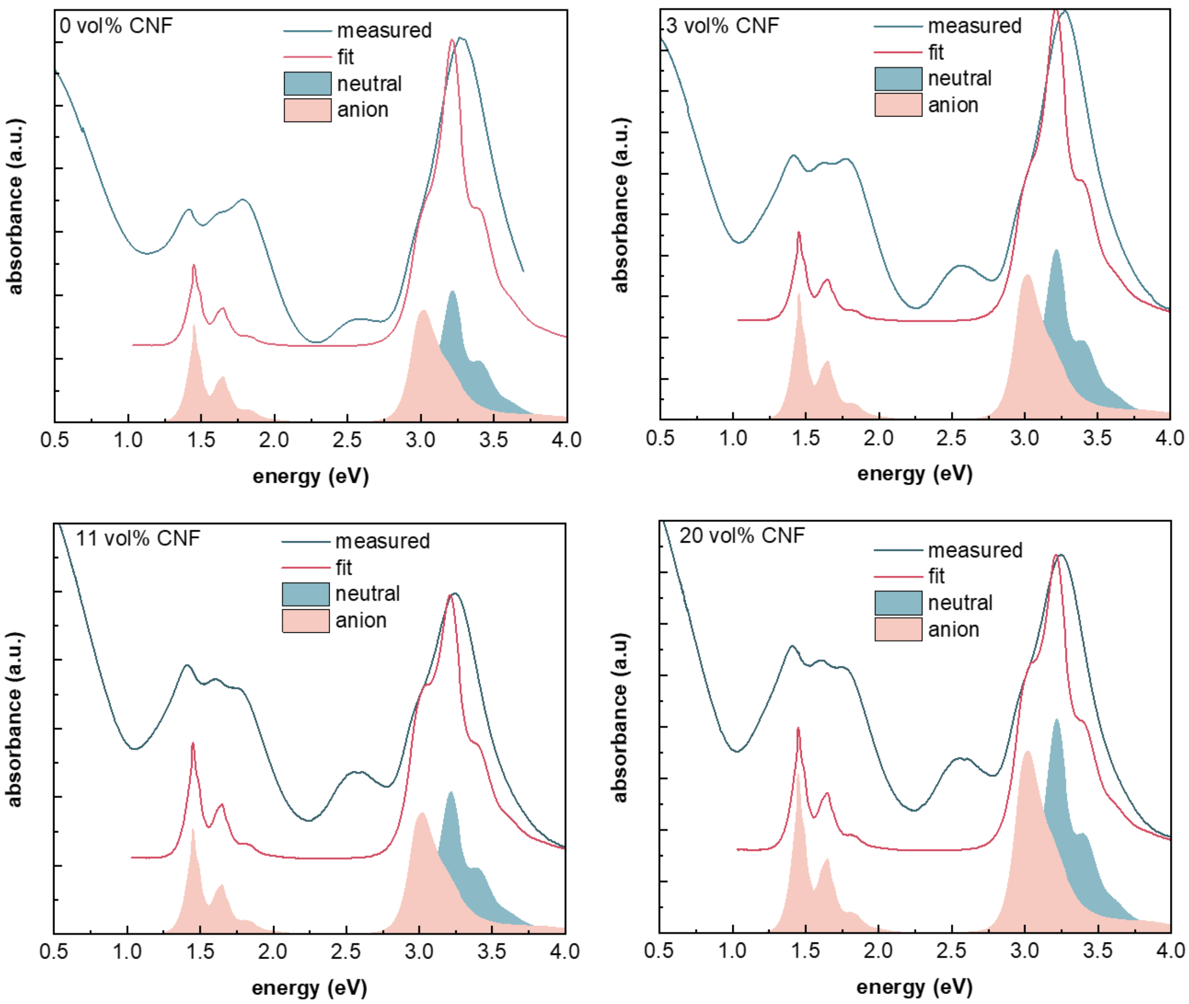

Figure S12. UV-vis spectra of nanocellulose films co-processed with 20 mol% F_4TCNQ (relative to the g_42T-T repeat unit) (blue solid line), of neutral F_4TCNQ and its anions in acetonitrile from Kiefer et al., *Nature Mater*. **2019**, *18*, 149-155 (blue and red filled curves) and fit of the measured spectra in the UV region composed of the spectra of neutral F_4TCNQ and its anion as well as a vertical offset.
